# Noninvasive Methods Unveil the Trophic Transmission of the Tapeworm *Ligula intestinalis* in Gull‐Billed Terns

**DOI:** 10.1002/ece3.70564

**Published:** 2024-11-13

**Authors:** Sofía Capasso, Manuel Parejo, José Manuel Reyes‐González, Juan G. Navedo, Ricardo Morán‐López, José A. Masero, Jorge S. Gutiérrez

**Affiliations:** ^1^ Departamento de Anatomía, Biología Celular y Zoología, Facultad de Ciencias Universidad de Extremadura Badajoz Spain; ^2^ Centro de Estudios Parasitológicos y de Vectores (CCT LaPlata‐CONICET‐UNLP) La Plata Argentina; ^3^ Conservation Biology Research Group Universidad de Extremadura Badajoz Spain; ^4^ Department of Renewable Marine Resources, Institut de Ciències del Mar (ICM‐CSIC) Barcelona Spain; ^5^ Bird Ecology Lab, Instituto de Ciencias Marinas y Limnológicas, Universidad Austral de Chile Valdivia Chile; ^6^ Ecología en el Antropoceno, Unidad Asociada CSIC‐UEX, Facultad de Ciencias Universidad de Extremadura Badajoz Spain

**Keywords:** Cestoda, fecal egg count, fish‐eating birds, helminths, mini‐FLOTAC, real‐time qPCR

## Abstract

Recent developments in microscopic and molecular tools have allowed the implementation of new approaches for assessing parasitic infections in wildlife populations. This is particularly important for the noninvasive detection and quantification of endoparasites in live animals. Here, we combined copromicroscopic (Mini‐FLOTAC) and molecular (qPCR) techniques to detect the infection of the macroparasite *Ligula intestinalis* (Cestoda, Pseudophyllidea) in fresh droppings of Gull‐billed Terns *Gelochelidon nilotica* (Charadriiformes, Laridae) breeding in southwestern Spain. Additionally, we sequenced the cytochrome *b* gene in parasite isolates from Gull‐billed Terns (definitive host) and Common Bleak *Alburnus alburnus* (second intermediate host) sampled around tern colonies to explore potential genetic differences between the isolates. The qPCR test showed a higher prevalence (18%; in 13/73 samples) than Mini‐FLOTAC (9%; in 8/88 samples), indicating that qPCR was more sensitive for diagnostic purposes than fecal flotation alone. Although the agreement between both techniques was substantial (84.2%) mainly due to the large number of uninfected samples, only Mini‐FLOTAC allowed us to quantify parasite shedding. When combining techniques, the prevalence of infection did not differ between adults and chicks, suggesting frequent trophic transmission from parents to their offspring via food provisioning. Phylogenetic analyses identified four haplotypes in the isolates from Gull‐billed Terns and Bleak, all of which were placed within a European clade composed of tapeworms recovered exclusively from phylogenetically derived cyprinid fish. This, combined with the short lifespan of mature tapeworms, suggests that Gull‐billed Terns became infected after consuming infected fish around their breeding colonies rather than on their West African wintering grounds. Altogether, our results represent the first record of *L. intestinalis* in Gull‐billed Terns and the first molecular characterization of the parasite in the Iberian Peninsula. This integrative coprodiagnostic protocol can be applied to other host–parasite systems, allowing researchers to study helminth infections in wild populations using a noninvasive approach.

## Graphical abstract

Using both copromicroscopic (Mini‐FLOTAC) and molecular (qPCR) techniques, we detected and quantified the macroparasite *Ligula intestinalis* in Gull‐billed Terns for the first time. Phylogenetic analyses revealed genetic similarities among parasite isolates from birds (definitive hosts) and fish prey (intermediate hosts), suggesting local infection from invasive Common Bleak. This integrative approach offers a noninvasive method for studying helminth infections in wild populations.

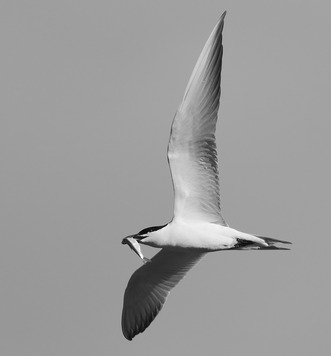

## 1 Introduction

Parasites can shape ecological interactions such as competition, mutualism, and predation, which ultimately influence population dynamics, community structure, and evolutionary processes (Johnson et al. 2019; Hasik et al. 2023). Some parasitic worms (i.e., helminths) complete their life cycle in a single host, while others are trophically transmitted, multihost parasites with a complex life cycle, in which one or more intermediate hosts are infected before transmission to a definitive or final host (Benesh, Parker, and Chubb 2021). These parasites are ubiquitous and can have negative or sublethal effects on host health and body condition (Weber et al. 2022). In particular, the tapeworm *Ligula intestinalis* (Linnaeus, 1758) (Cestoda: Diphyllobothridae) is an ideal model to study these effects at the molecular, organismal, and population levels (Hoole, Carter, and Dufour 2010). It is a worldwide‐distributed generalist parasite with a complex life cycle involving a planktonic copepod as the first intermediate host, a wide range of freshwater fishes as the second intermediate host, and a fish‐eating bird as the definitive host (Hoole, Carter, and Dufour 2010; Scholz and Kuchta 2016; Kuchta and Scholz 2017; Gutiérrez and Hoole 2022). The larval stage (or plerocercoid) can infect more than 170 fish species from different genera and is the causative agent of ligulosis disease, which is associated with abdominal distortion, immune responses, gonad atrophy, and negative effects on the energy balance of the host (Gutiérrez and Hoole 2022). Notably, *L. intestinalis* can play an important role in driving the population dynamics of wild fish and cause severe losses in pisciculture (Kennedy, Shears, and Shears 2001; Gutiérrez and Hoole 2022). However, current knowledge of its host range and disease severity is more limited in definitive hosts.

**FIGURE 1:**
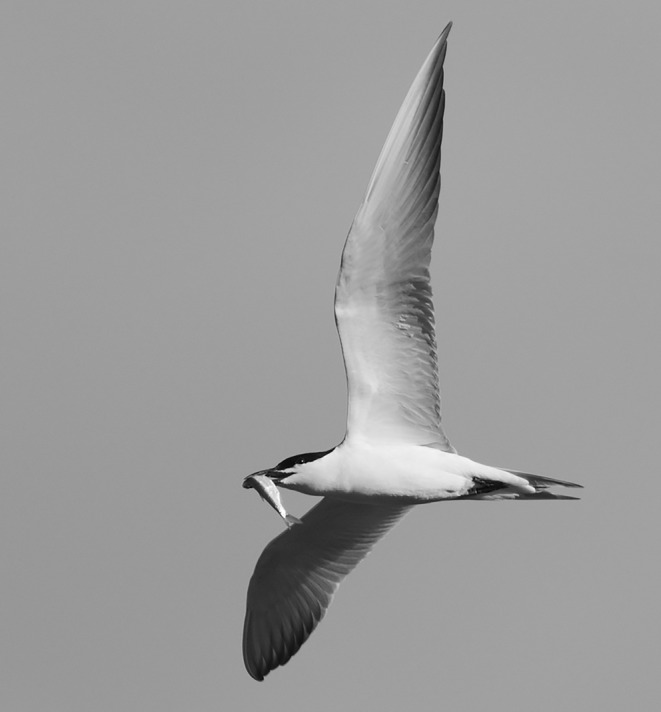
Gull‐billed Tern preying on Bleak within the study area. Credit: Atanasio Fernández García.

According to the global database of helminth–host parasite records, *L. intestinalis* has been recorded in 55 bird species globally (Gibson, Bray, and Harris 2005). Although it is a widespread parasite of fish‐eating birds across Europe, infections in the Iberian Peninsula have only been recorded at the bird genus level: *Podiceps* spp., *Mergus* spp., *Phalacrocorax* spp., and *Sterna* spp. (Cordero del Campillo, Castañón Ordóñez, and Reguera Feo 1994). Among terns, the Gull‐billed Tern ( *Gelochelidon nilotica* [Gmelin, 1789], Figure 1), a summer migrant breeding in the Iberian Peninsula, is a candidate (yet unreported) definitive host. In fact, Gull‐billed Terns are generalist and opportunistic feeders consuming a variety of terrestrial and aquatic animals (Molina et al. 2023), including intermediate fish hosts such as the Common Bleak ( *Alburnus alburnus* [Linnaeus, 1758], Figure 1), a limnophilic cyprinid native to most of Europe but invasive in the Iberian Peninsula (Almeida et al. 2017; Latorre et al. 2023). However, there is little information about the extent and significance of such infections in definitive avian hosts since the pioneering work of Dubinina (1980).

The study of helminth infections in their hosts is often limited by difficulties in detecting and measuring endoparasite burdens. Traditionally, helminth infections have been routinely assessed by necropsy. This method allows direct counts of helminths and is therefore a reliable indicator of parasite burden but may not be viable for hosts of conservation importance or for longitudinal studies. Alternatively, identifying helminth parasites from live animals in a noninvasive manner represents an ongoing methodological challenge for wildlife parasitologists and ecologists. For ethical reasons, it is important to avoid stressful and destructive sampling, especially when host species are protected or endangered (Rojas et al. 2024). In the last decades, different noninvasive techniques have been employed for the detection of helminth parasites in wild bird populations (e.g., Burthe et al. 2013; Lobos‐Ovalle et al. 2021). For example, endoscopy has facilitated the study of natural burdens of nematodes in free‐ranging European shags (*Gulosus aristotelis* [Linnaeus, 1761]) (Burthe et al. 2013). Nevertheless, the use of endoscopy is limited if the parasites and/or hosts are small‐sized or if the parasites of interest are located lower down than the stomach in the gastrointestinal tract (Burthe et al. 2013). A more common, still indirect technique used for the detection and quantification of helminths in wild animal populations is based on fecal egg counting (Newbold et al. 2017; Nielsen 2021). Despite some limitations (such as density‐dependent worm fecundity, temporal variation in egg shedding rates, and poor sensitivity at low worm burdens), fecal egg counts are often the only available method for quantifying individual parasite burdens without destructive sampling (Newbold et al. 2017; Papaiakovou, Gasser, and Littlewood 2019; Nielsen 2021). The Kato–Katz and McMaster techniques are commonly used diagnostic methods in public and animal health; however, newer quantitative coprological techniques such as Mini‐FLOTAC generally offer greater sensitivity, particularly for low‐intensity infections (Bondarenko et al. 2009; Cringoli et al. 2017). Mini‐FLOTAC allows the simultaneous diagnosis of helminth eggs/larvae and protozoa oocysts/cysts, offering an advantage over the other techniques. In addition, it can be performed on either fresh or fixed fecal samples, meaning that samples can be processed days or weeks after transfer to the laboratory (Cringoli et al. 2017).

More recently, new molecular techniques are becoming increasingly popular for the detection and surveillance of parasites (Bass et al. 2015). Notably, genetics and genomics offer the possibility of nonlethal sampling plus fast accurate parasite identification and genetic characterization. In doing so, they can help overcome difficulties in identifying cryptic parasite species, ultimately resolving phylogenetic relationships (Huggins et al. 2017; Bass et al. 2023). Previous phylogenetic studies suggest that *L. intestinalis* may in fact be composed of several lineages or cryptic species with different hosts and geographic distributions (Olson and Tkach 2005; Bouzid et al. 2008; Nazarizadeh et al. 2023). This makes this parasite a good model for studying speciation and the evolution of parasite genetic diversity (Hoole, Carter, and Dufour 2010).

The objectives of the present study were threefold. First, to assess the potential infection of *L. intestinalis* in Gull‐billed Terns in a noninvasive and minimally stressful manner. To do this, we combined copromicroscopic (Mini‐FLOTAC) and molecular (qPCR) techniques to detect *L. intestinalis* in fresh droppings of Gull‐billed Tern adults and chicks in southwestern Spain. Our second aim was to compare the detection capability and practical application of the two protocols. Finally, we provide the first molecular characterization of the parasite for the Iberian Peninsula using isolates from naturally infected Gull‐billed Terns and Common Bleak.

## 2 Materials and Methods

### 2.1 Study Area and Sampling

**FIGURE 2:**
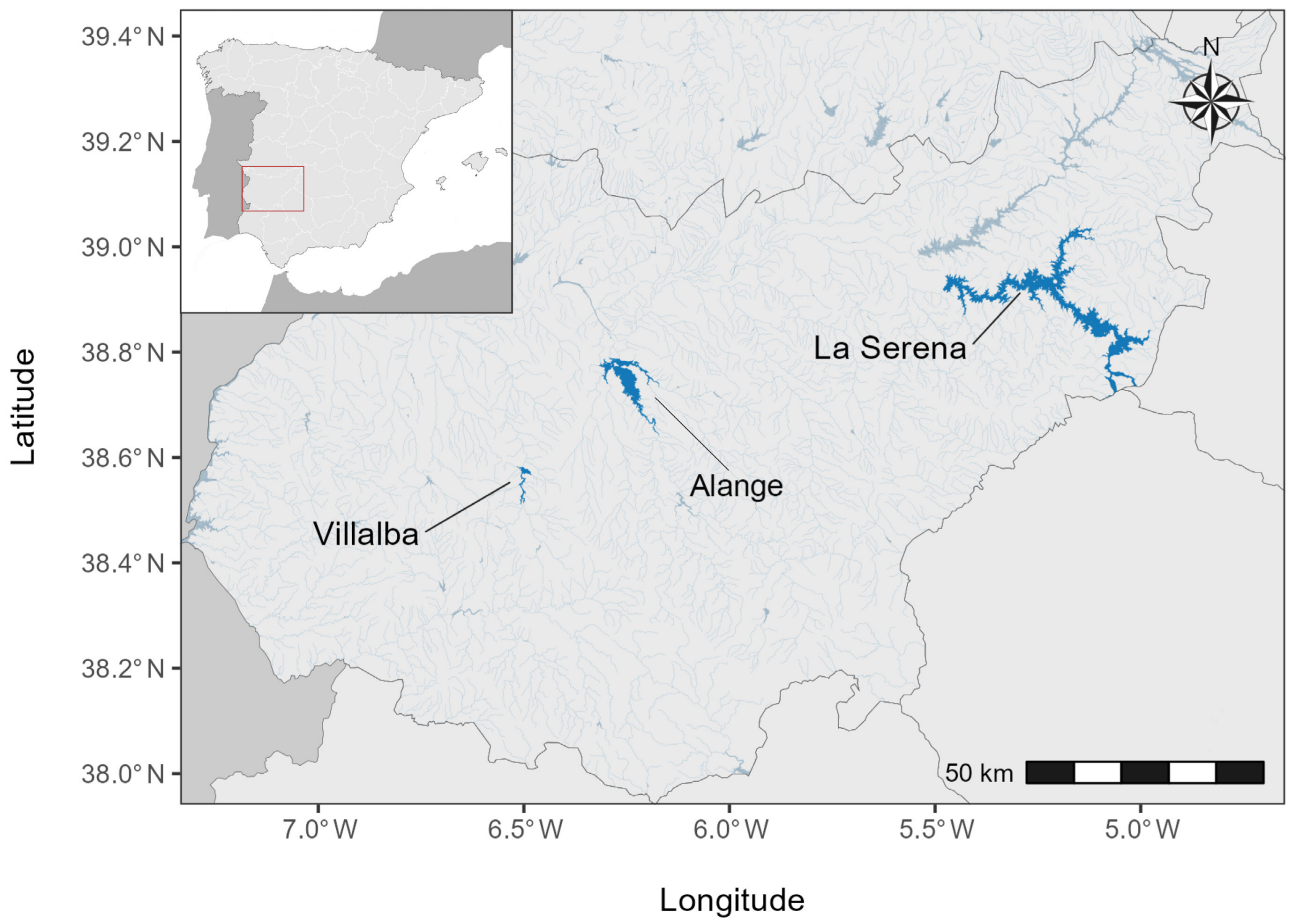
Map of the study sites in the province of Badajoz (Extremadura, Spain) showing sampling localities.

We conducted the study during two consecutive breeding seasons (May–July 2022 and 2023) in three Gull‐billed Tern nesting colonies located within the Guadiana basin in Extremadura, southwestern Spain: Alange, Villalba, and La Serena (Figure 2). About 1000 Gull‐billed Tern pairs breed regularly on islands situated in large reservoirs (Corbacho, Sánchez‐Guzmán, and Villegas 2009; Villegas et al. 2013).

The colonies were monitored regularly (every 3–4 days) throughout the breeding season, from the start of egg laying (mid‐May). Surveys were carried out in the early morning to avoid heat stress and minimize potential circadian variation in egg excretion by *L. intestinalis* . During surveys, we searched for new nests, recorded the status of previously marked nests, and searched for chicks (for details, see Abad‐Gómez et al. 2012; Villegas et al. 2013). Birds were caught using clap‐net traps (adults) or by hand (chicks). Upon capture, birds were individually placed into foldable fabric boxes (22 × 34 × 16 cm), whose floor was covered with a plastic tray. A wire mesh platform was placed 2 cm above the plastic tray to allow individuals to stand without touching the tray and excreta. Freshly released droppings were immediately collected with a disposable plastic spoon and transferred into a tube labeled with an individual code. Then, droppings were weighed, preserved in 70% ethanol, and stored at 4°C until further analysis. A total of 88 droppings were collected from adults and chicks (57 and 31, respectively). Adults were measured and marked with metal rings, while chicks were measured and marked with individually numbered plastic bands that were later replaced by metal rings. Sex was determined in both adults and chicks sampled on their nest by PCR amplifications of the CHD genes following the protocol of Lee et al. (2010).

In parallel, we sampled invasive Common Bleak (a second intermediate host for *L. intestinalis* ; see above) using gillnets in Alange and Villalba. Fish were immediately euthanized by immersion in an overdose solution of clove oil (0.05 mL/L) or by rapid chilling in an ice bath. The specimens were stored in ice during transport to the laboratory, where they were dissected to check for the presence of *L. intestinalis* plerocercoids. Plerocercoids found were preserved in 70% ethanol and stored at 4°C until molecular analysis (see below).

All procedures were approved by the Ethics Committee of the University of Extremadura (license 112//2020) and the Government of Extremadura (license lliCN0001/23/ACA).

### 2.2 Morphological Identification of *L. intestinalis*

Bird dropping samples were analyzed to determine parasite egg counts using the Mini‐FLOTAC method (Cringoli et al. 2017). Approximately 1 g of the dropping sample (0.7 ± 0.3 g) was placed in the Fill‐FLOTAC and then diluted and homogenized with 9 mL of a zinc sulfate flotation solution (specific gravity = 1.35; dilution ratio 1:10). One milliliter of the filtered fecal suspension was transferred from the Fill‐FLOTAC to the two Mini‐FLOTAC reading disk chambers. After 10 min, the samples were analyzed under a microscope (Nikon Eclipse 50i, Nikon Instruments Inc., Melville, USA) at 40× magnification. Egg counts of each sample were expressed as eggs per gram (EPG) of dropping, using a multiplication factor of 5 (Cringoli et al. 2017). Eggs were photographed using a light microscope at 20× and 40× magnifications and measured using ImageJ software (Schneider, Rasband, and Eliceiri 2012).

### 2.3 Molecular Identification of *L. intestinalis*

#### 2.3.1 DNA Extraction and PCR

First, we used 23 *L. intestinalis* plerocercoid larvae to optimize and determine the DNA extraction method. A 0.3–0.5‐cm section of each larva was cut and placed in 150 μL of digestion buffer (NaCl 100 mM, tris/HCl 10 mM, EDTA 25 mM, pH 8.4) with 0.01% of SDS and left overnight with agitation at 56°C. Then, we extracted the DNA with the MagMAX Viral/Pathogen Nucleic Acid Isolation Kit reagent. Finally, we eluted in 50 μL of H_2_O and measured the DNA concentration after extraction using a NanoDrop2000c UV–vis spectrophotometer (NanoDrop Technologies, Wilmington, USA). Cytochrome *b* was amplified using COBA–COBB primers (Bouzid et al. 2008). PCRs were carried out in 20 μL using 100 ng of extracted DNA, 400 pM of each primer, 4 μL of MyTaq reaction buffer 5x, and 0.625 U of MyTaq polymerase (Bioline). The PCR cycles' parameters were the following: initial denaturation for 3 min at 94°C; 40 cycles of 10 s at 98°C, 30 s at 50°C, and 1 min at 72°C; and a final extension step for 10 min at 72°C. A thermal cycler (Veriti 96‐well Thermal Cycler, ThermoFisher, Waltham; Massachusetts, EEUU) was used to perform PCR, and the PCR products were separated using 1% agarose gel electrophoresis. Contiguous sequences were run in 3500 Series Genetic Analyzers (ThermoFisher, Waltham; Massachusetts, EEUU) using the integrated 3500/3500xL Data Collection Software.

Next, we used aliquots of 84 dropping samples (out of 87 collected) for molecular analysis (45 from adults and 39 from chicks). In the case of chicks, samples came from 10‐day‐old individuals or older since parasite eggs first appear in the uterus of the tapeworm 45–50 h after infection (Dubinina 1980) and thus infections are not likely to be detected in the first days after hatching. The DNA extraction protocol for the droppings was similar to that used for plerocercoids (see above), using 100 μL of dropping per sample and the same primers. To obtain a homogeneous suspension, we resuspended the samples with a pipette. We transferred approximately 100 μL to a new tube containing 150 μL of commercial digestion buffer (ThermoFisher) supplemented with 15 μL of proteinase K 20 mg/mL. Then, the samples were stored overnight at 56°C for 16–18 h with continuous shaking. The next day, this suspension was used for extraction using the MagMAX Pathogen Kit, which consists of chemical and mechanical lysis. Then, qPCRs were carried out in 25 μL containing Takara Mastermix SYBRGreen (2x) plus 0.5 μL of ROX (Takara Bio Inc. Dalian, China), 1 μL of extracted DNA solution, 0.2 μM of each primer, and 10 μL of sterile double‐distilled water (ddH_2_O). The conditions for PCR were the following: initial denaturation for 5 min at 95°C; 40 cycles of 15 s at 95°C, 30 s at 58°C, and 1 min at 72°C. Finally, it performed a melt curve stage (ramp up 2.63°C/s to 95°C and stand 15 s, then ramp down 2.42°C/s to 60°C and stand 1 min, and then ramp up 0.05°C/s to 95°C and stand 15 s). A thermal cycler (QuantStudio 6 Flex, ThermoFisher, Waltham; Massachusetts, EEUU) was used to perform qPCR. The genomic DNA previously extracted from *L. intestinalis* was used as a positive control in each PCR run. Negative controls (template replaced with ddH_2_O) were included in runs to check for potential contamination.

We concluded that a bird was infected if the sample yielded amplification of the studied gene (COBA/COBB) and the value of its melting temperature was identical to that detected in positive control samples. These results were corroborated by the sequence of the fragments amplified in the qPCR. The new cytochrome *b* sequences were deposited in GenBank under the corresponding accession numbers.

#### 2.3.2 Phylogenetic Analyses

We conducted a phylogenetic analysis using the newly obtained sequences of the cytochrome *b* along with matching sequences in GenBank of *L. intestinalis* founded in fish following the last phylogeny of the species complex (Nazarizadeh et al. 2023) (Table A1). For phylogenetic tree reconstruction under maximum likelihood (MI), DNA sequences were aligned by CLUSTALW using MEGA version 10 (Kumar et al. 2018). The alignments were trimmed to the length of the shortest sequence. The best‐fit model of DNA sequence evolution was selected using jModeltest 2.1.7 (Darriba et al. 2012). According to the Akaike information criterion, we used the Hasegawa–Kishino–Yano including variation among sites (HKY+G). Phylogenetic reconstruction was performed with BEAST 1.8.4. (Drummond et al. 2012). Tree priors were selected using the interface BEAUTi 1.8.4. (Drummond et al. 2012) with a strict clock and a Yule speciation process. Markov chain Monte Carlo (MCMC) simulations were run with 5,000,000 generations, and one tree was recorded every 1000 generations. In all, 10% of the trees were discarded as burn‐in in TreeAnnotator (BEAST software). We validated the results of the Bayesian analyses in Tracer 1.6. (Drummond and Rambaut 2007). Phylogenetic relationships under Bayesian inference (BI) were also generated in MrBayes v3.2.7 (Ronquist et al. 2012). Two independent runs were performed for 10,000,000 generations and sampled every 100th generation. The burn‐in was set for the first 25% of the sampled trees. The phylogenetic tree was constructed with FigTree 1.4.3 (Rambaut 2007). DnaSP version 6.0 (Librado and Rozas 2009) was used to estimate nucleotide diversity, haplotype diversity, and sequence polymorphisms.

### 2.4 Statistical Analyses

We calculated three key infection indices following Reiczigel et al. (2019): (1) prevalence: the fraction of parasitized individuals in the host population; (2) mean intensity: the average EPG across infested hosts in the population; and (3) mean abundance: the average EPG across all host individuals, either infected or noninfected.

To assess the potential effects of age, sex, and site on infection indices, we fitted generalized linear models (GLMs) using the package “lme4” (Bates et al. 2015) in the R environment version 4.2.3 (R Core Team 2022). We explored model residuals using a simulation‐based approach to create readily interpretable scaled (quantile) residuals for the fitted GLM (Hartig 2022).

To test whether sex and site influenced infection indices, we ran two GLMs using a reduced dataset that included data from adults only, all of which were molecularly sexed. We first ran a GLM with a binomial error distribution and a logit link function including infection status (parasitized vs. nonparasitized, combining results from qPCR and Mini‐FLOTAC techniques) as the response variable and sex and site as predictor variables (model 1). Second, we fitted a GLM with a negative binomial distribution including EPG as the response variable and sex and site as predictor variables (model 2). We conducted multiple Tukey post hoc comparisons using the package “multcomp” (Hothorn, Bretz, and Westfall 2008) to compare the prevalence of infection and EPG between sites (i.e., colonies).

To evaluate differences in infection between adults and offspring, we fitted a GLM with infection status (parasitized vs. nonparasitized) as the response variable and host age (adult vs. large chick) as the predictor variable, with a binomial distribution and a logit function (model 3). To maximize sample size in this model, we did not test for potential sex‐specific differences in infection since we only collected blood samples from newly hatched birds for molecular sexing and therefore we did not know the sex of large chicks captured outside their nests. We did not include site in this model as chick (10‐day‐old or older) dropping samples came almost exclusively from Villalba. Then, we fitted a GLM with a negative binomial distribution, including EPG as the response variable and host age as the predictor variable (model 4).

Finally, we estimated the agreement in the detection rate of positive samples between qPCR and Mini‐FLOTAC techniques testing by Cohen's Kappa coefficient (Cohen 1960) using the “irr” package (Gamer, Lemon, and Fellows 2019). We considered *p*‐values < 0.05 to be statistically significant.

## 3 Results

### 3.1 Infection Indices Through Mini‐FLOTAC

According to the microscopic examination, 8/88 samples (9.09%) were positive for *L. intestinalis* . Eggs were ovoid‐shaped with mean size 62.34 μm (52.88–76.19) × 42.03 μm (38.11–50.70), with the characteristic polar cap in one extreme (Figure A1).

**FIGURE 3:**
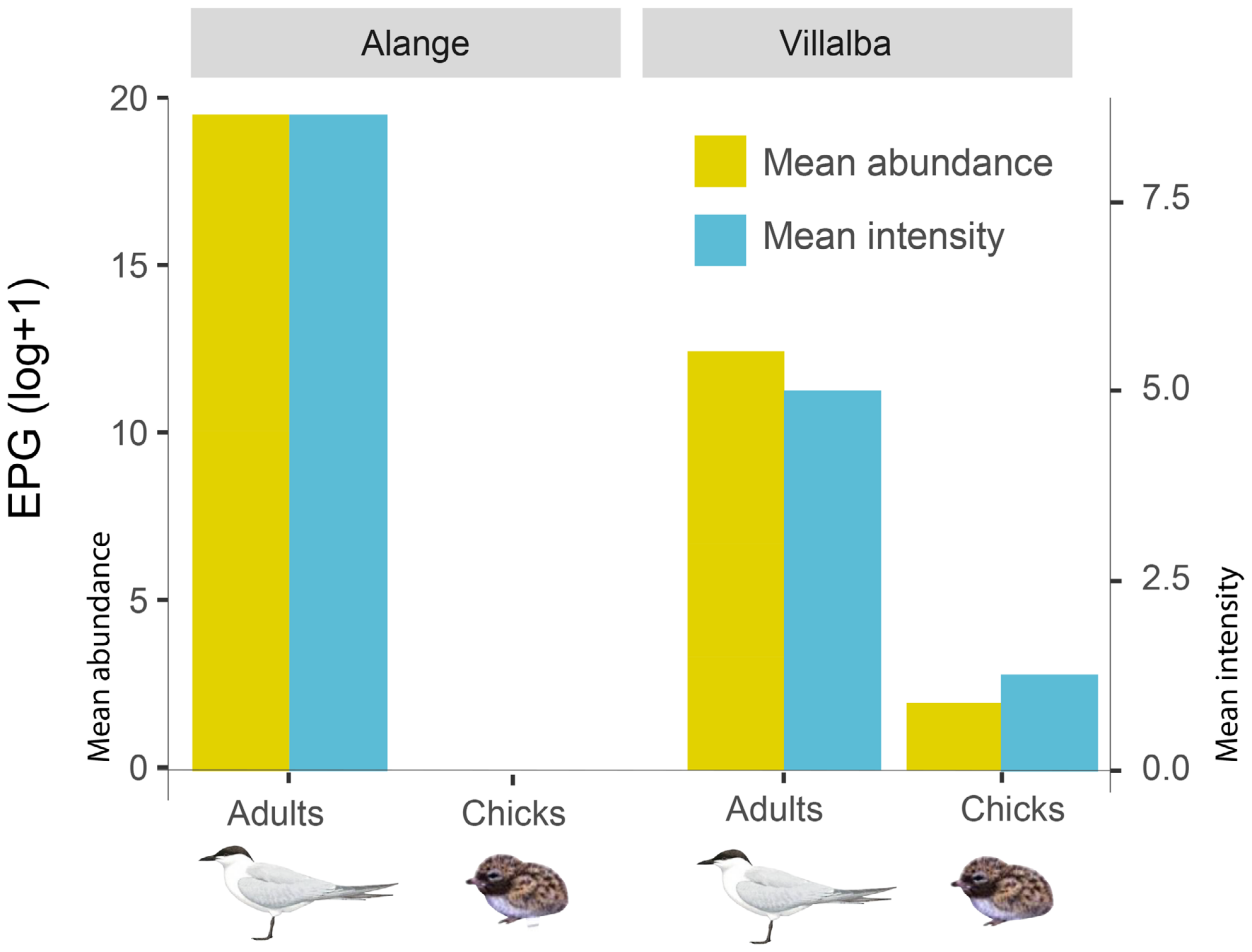
Mean abundance and mean intensity of EPG in chicks and adults at each sampling site. Only sites with positive cases are shown.

In adults, we found no differences in the prevalence between sexes (model 1: estimate = 0.47, *z* = 0.65, *p* = 0.51) or colonies (post hoc test: *z* = −0.28, *p* = 0.95). However, there were differences in the abundance of infection between colonies, being lower in Villalba than in Alange (model 2: estimate = −6.54, *z* = −2.74, *p* = 0.006; post hoc test: *z* = −2.74, *p* = 0.012; Table 1; Figure 3). We found no differences in parasite abundance between sexes (model 2: estimate = 0.67, *z* = 0.28, *p* = 0.77). No birds in La Serena were parasitized by *L. intestinalis* .

**TABLE 1:** Prevalence, mean abundance, intensity, and range of *L. intestinalis* (based on counts of EPG in flotation with Mini‐FLOTAC) in Gull‐billed Terns sampled in Extremadura, Spain, and Iberian Peninsula.

Site	Age	No. samples	Prevalence^a^	Mean abundance EPG ± SE	Mean intensity EPG ± SE	Range EPG (min–max)
Alange	Adults	22	27.3%	1332 ± 5635	9325 ± 8280 (*n* = 3)	0–25,840
	Chicks	1	—	0	0	0
Villalba	Adults	25	24%	26.2 ± 122	158 ± 148 (*n* = 4)	0–600
	Chicks	27	18.5%	0.2 ± 0.91	5 (*n* = 1)	0–5
La Serena	Adults	10	—	0	0	0
	Chicks	3	—	0	0	0
Pooled	Adults	57	21.1%	520 ± 3490	4086 ± 9624 (*n* = 7)	0–25,840
	Chicks	31	16.1%	0.2 ± 0.91	5 (*n* = 1)	0–5

^a^ Prevalence includes positives from PCR and Mini‐FLOTAC.

### 3.2 Infection Status Through qPCR

According to molecular detection, 13/73 samples (17.8%) were positive for *L. intestinalis* . Unlike Mini‐FLOTAC (see above), the qPCR technique detected *L. intestinalis* in most large chicks (12.9%; in 4/31 samples). BLAST similarity analysis confirmed the correct amplification of the mitochondrial marker and the classification of the isolates from fish and birds into the species *L. intestinalis* . The length of cytochrome *b* sequences obtained was 399 bp for the isolate from fish (GenBank Acc. No. PP781512) and 402 bp from birds (GenBank Acc. No. PP781513‐PP781515). In total, we found four haplotypes, with eight variable (polymorphic) sites between them (site positions: 56, 112, 127, 137, 151, 178, 191, and 253).

**FIGURE 4:**
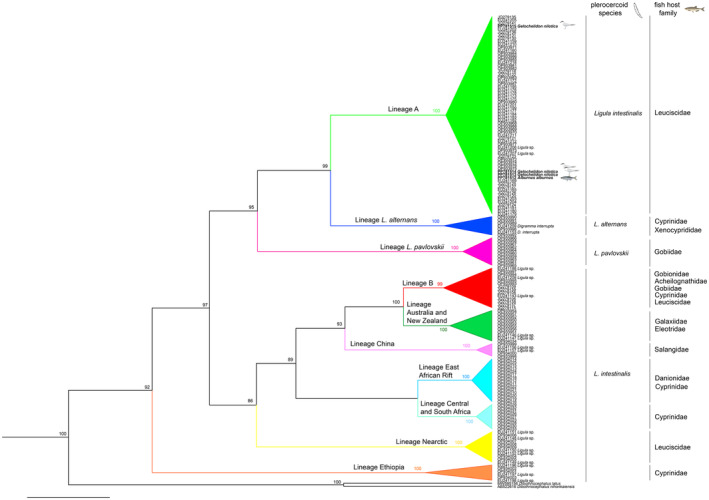
Phylogenetic relationship between *Ligula intestinalis* and different fish host families, as inferred from sequences of cytochrome *b* analyzed by Bayesian inference (BI) and maximum likelihood (ML) methods. Branch support values at internodes show the posterior probabilities/bootstrap percentage from BI and MI analyses, accordingly.

BI and ML analyses of the cytochrome *b* gene generated a consistent tree with a well‐supported phylogenetic structure (Figure 4). The isolates from fish and birds obtained in this study correspond to the lineage A (European and North African populations) of the *L. intestinalis* species complex (see Bouzid et al. 2008; Nazarizadeh et al. 2023). The *L. intestinalis* representatives included in our analyses formed a monophyletic group with a clear geographical or host‐related pattern (European‐derived cyprinids).

### 3.3 Combination of “Classical” and Modern Methods

Overall, combining qPCR and Mini‐FLOTAC techniques, we detected *L. intestinalis* in 17/88 dropping samples (19.3% prevalence). Prevalence did not differ between adults (21.1%, 12/57) and chicks (16.1%, 5/31) (model 3: estimate = −0.69, *z* = −0.84, *p* = 0.4; Figure 3). However, the EPG mean abundance was lower in chicks than in adults (model 4: estimate = −7.64, *z* = −3.30, *p* = 0.0009).

**FIGURE 5:**
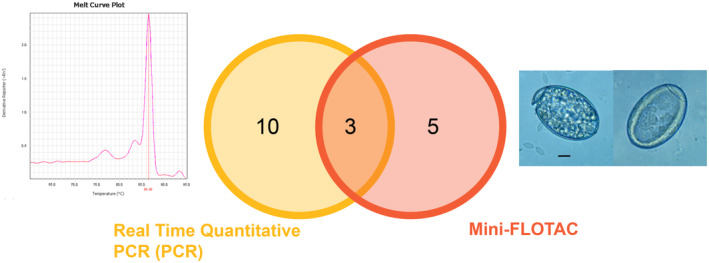
Positive samples detected by each methodology and by both methodologies (in the intersection). On the left side, melt curve analysis from the qPCR assay with a *L. intestinalis* DNA template is shown. On the right side, eggs with a polar cap observed after flotation with Mini‐FLOTAC are observed. Scale bar = 25 μm.

The qPCR method detected twice as many positive samples than Mini‐FLOTAC (Figure 5), and only two samples identified as positive by Mini‐FLOTAC were undetected by qPCR. The agreement between both techniques was substantial (84.2%, *κ* = 0.38) and greater than would be expected by chance (*z* = 3.11, *p* = 0.001). When considering positive cases only, the agreement (30.8%; *κ* = −0.31) was lower than would be expected by chance (*z* = −1.66, *p* = 0.09).

## 4 Discussion

By combining microscopic and molecular techniques, we detect for the first time *L. intestinalis* in fresh droppings of Gull‐billed Terns. The use of qPCR allowed us to successfully amplify parasite DNA from dropping samples after parasite egg rupture, while the Mini‐FLOTAC technique also permitted the quantification of parasite eggs. This study contributes to the large yet incomplete range of bird species that the parasite has been recorded from and provides an integrative noninvasive approach for future studies on wild bird populations.

The application of molecular techniques allowed us to find important ecological features of this host–parasite interaction. Notably, qPCR demonstrated that *L. intestinalis* can be detected in chick droppings. In the reservoirs studied, Gull‐billed Terns fed extensively on Common Bleak (Figure 1) among other prey (authors' unpublished data); therefore, we expected adults to act as sources of infection for their chicks, as the chicks are unable to feed themselves (they are fed by both parents, though most often by the female; Lind 1963). Indeed, we found *L. intestinalis* in chick droppings as early as 10–11 days after hatching, suggesting frequent trophic transmission from parents to their chicks via food provisioning. The plerocercoid larvae, provisioned together with small‐infected fish, developed into mature worms in the intestines of chicks (parasite shedding occurs after 4–5 days; Dubinina 1980). Infections in early life could be promoted by the higher incidence of infection in younger Common Bleak that can be swallowed whole by chicks (Harris and Wheeler 1974). The provision of infected prey by parents could result in a similar or even higher parasite burden in chicks than in adults. For example, the prevalence of the nematode *Stegophorus macronectes* in Chinstrap Penguins ( *Pygoscelis antarctica* ) from Deception Island (South Shetlands, Antarctica) was higher in chicks than in adults (Vidal et al. 2012). This could be explained by the less developed immunity in chicks, the regurgitation of infective larvae by parents, or even the transfer of adult parasites living in the gastrointestinal tracts of their parents (Muzaffar and Jones 2004; Vidal et al. 2012). Although birds show behavioral adaptations to avoid consuming parasitized prey (Bush and Clayton 2018), little is known about whether trophically transmitted parasites reduce host fitness and whether the impact of parasitism is similar among family members (Granroth‐Wilding et al. 2015). Along this line, Patton et al. (2017) reported a mass die‐off of Gull‐billed Terns breeding in California, USA, caused by peritonitis attributable to the acanthocephalan *Profilicollis altmani* . Further studies are thus required to investigate the direct and indirect effects of helminthic infection on parents and offspring and ultimately to better understand the cost–benefit balance of consuming trophically transmitted parasites (Lafferty 1999).

Furthermore, the phylogenetic analysis based on cytochrome *b* data corroborates the inclusion of present specimens into *L. intestinalis* , placing the new haplotypes in the “clade A” of the species complex (Bouzid et al. 2008). The parasite samples of clade A (European, Russian, and North African populations) come from phylogenetically derived cyprinid fish (*Abramis*, *Alburnus*, *Phoxinus*, *Rutilus*, *Scardinius*), whereas the clade B (European and North African populations) parasitize basal cyprinid species (*Barbus*, *Gobio*, *Rhodeus*). Several population genetic studies of *L. intestinalis* found a hidden genetic variability, indicating the possible existence of cryptic lineages (Bouzid et al. 2008; Štefka, Hypša, and Scholz 2009; Nazarizadeh et al. 2023). These studies have demonstrated a geographically dependent pattern in the genetic structure of the *L. intestinalis* species complex (Bouzid et al. 2008; Štefka, Hypša, and Scholz 2009; Nazarizadeh et al. 2023). On a broad geographic scale, this pattern is affected by long‐distance transmissions mediated by introduced fish and/or migrating birds (Bouzid et al. 2008). Both are criteria met in our study system, since the Common Bleak is an invasive host in the Iberian Peninsula (Latorre et al. 2023) and the Gull‐billed Tern is a long‐distance migratory species.

Moreover, it has been hypothesized that the feeding strategy of derived and pelagic (clade A) versus basal and benthic cyprinid fish (clade B) represents a potential diversifying force in the *L. intestinalis* species complex (Bouzid et al. 2008). Considering that Gull‐billed Terns catch fish from the water surface, our results support this view. The haplotypes found in the bird droppings, the fact that the birds had been nesting for several weeks when they were captured, and the relatively short lifespan of mature tapeworms in the intestines of the final host (up to 7 days in the Great crested Grebe *Podiceps cristatus* ; Dubinina 1980) indicate that all birds acquired the parasites locally while feeding around their breeding colonies rather than on their West African wintering grounds or *en route* toward breeding areas. In addition, differences in parasite abundance among sites are probably due to the higher incidence of infestation of Common Bleak in Alange during the study period (authors' unpublished data).

Although the two screening methods detected *L. intestinalis* , the positive cases detected by qPCR were almost twice as many as with Mini‐FLOTAC. This indicates a higher sensitivity in the infection detection of qPCR. The substantial agreement between both techniques (84.2%) probably was due to the large number of cases (57) assigned negative by the two methods. Discrepancies between methods could be explained by several reasons, including differences in the amount of parasite eggs/DNA in the aliquots (particularly at low parasitemias) or the choice of the flotation solution. Generally, morphological tests used in coproparasitology are imperfect, as no single method performs best for every parasite group. Moreover, the diagnostic sensitivity of standard coproparasitological methods is unknown for many parasite–host systems. The Mini‐FLOTAC technique was used here as a reference test to predict the prevalence and shedding intensity of *L. intestinalis* . It is important to note that differences in shedding patterns (Villanúa et al. 2006; de la Peña et al. 2024) and flotation procedures using different flotation solutions (Lobos‐Ovalle et al. 2021) could influence the recovery of parasite eggs. The Mini‐FLOTAC technique has been tested in wild birds, and the saturated zinc sulfate solution resulted in a significantly higher detection rate and higher fecal egg counts of gastrointestinal helminths (nematode, cestode, and trematode parasites) than the saturated salt solution (Lobos‐Ovalle et al. 2021). Indeed, cestode eggs were only identified by the zinc sulfate solution (Lobos‐Ovalle et al. 2021). Even though the accuracy and precision of the egg detection are often correlated with the volume of feces examined (Torgerson, Paul, and Lewis 2012), we did not find a positive relationship between sample volume and egg detectability (Figure A2); this indicates that Mini‐FLOTAC was a reliable alternative for detecting cestode infections in relatively small samples.

Likewise, molecular tools are not perfect and can lead to major gaps in parasitological research (Scholz 2024). One important limitation of molecular diagnostics is finding specific primers to successfully perform qPCR. Specific primers have been designed for the detection of *L. intestinalis* (Li, Liao, and Yang 2000; Logan et al. 2004; Bouzid et al. 2008), but these have only been used on *Ligula* isolates (plerocercoids) from various fish hosts. To successfully extract and amplify parasite DNA from droppings, samples require crucial steps (homogenization, agitation, a suitable extraction kit) to reduce interference and allow the rupture of the eggs. Although the qPCR technique detected a higher parasite prevalence compared to the flotation method, the molecular protocol may be improved by adding additional molecular markers to increase the accuracy of the results. Meanwhile, the combination of methodologies is currently the best approach for better diagnostic sensitivity. Importantly, this can minimize some of the obstacles associated with noninvasive parasitological surveys in wildlife populations (Schilling, Mazzamuto, and Romeo 2022).

Together, our results represent the first record of *L. intestinalis* in Gull‐billed Terns worldwide and the first molecular characterization of the parasite for the Iberian Peninsula. Although qPCR was more sensitive for diagnostic purposes than Mini‐FLOTAC, the latter allowed parasite quantification, highlighting the importance of combining “classical” and modern methods to better understand the complexity of parasites (Scholz 2024). This integrative, noninvasive approach can be applied to other host–parasite systems, helping researchers study helminth infections in wild populations.

## Author Contributions

**Sofía Capasso:** conceptualization (lead), data curation (lead), formal analysis (lead), investigation (lead), methodology (lead), writing – original draft (lead), writing – review and editing (lead). **Manuel Parejo:** methodology (equal), writing – review and editing (equal). **José Manuel Reyes‐González:** formal analysis (supporting), methodology (equal), writing – review and editing (equal). **Juan G. Navedo:** methodology (supporting), writing – review and editing (equal). **Ricardo Morán‐López:** methodology (supporting), writing – review and editing (equal). **José A. Masero:** methodology (supporting), writing – review and editing (equal). **Jorge S. Gutiérrez:** conceptualization (lead), formal analysis (equal), funding acquisition (lead), investigation (lead), methodology (equal), project administration (lead), supervision (lead), writing – original draft (lead), writing – review and editing (lead).

## Conflicts of Interest

The authors declare no conflicts of interest.

## Data Availability

Data supporting the results are available in Dryad Digital Repository: https://doi.org/10.5061/dryad.h70rxwdv0.
